# Exploring the Treasure of Plant Molecules With Integrated Biorefineries

**DOI:** 10.3389/fpls.2019.00478

**Published:** 2019-04-16

**Authors:** Andres F. Torres, Xuan Xu, Constantinos V. Nikiforidis, Johannes H. Bitter, Luisa M. Trindade

**Affiliations:** ^1^ Plant Breeding, Wageningen University and Research, Wageningen, Netherlands; ^2^ Biobased Chemistry and Technology, Wageningen University, Wageningen, Netherlands; ^3^ Graduate School Experimental Plant Sciences, Wageningen University, Wageningen, Netherlands; ^4^ Colegio de Ciencias Biológicas y Ambientales, Universidad San Francisco de Quito, Quito, Ecuador

**Keywords:** biorefinery, plant compounds, process technology, biomass deconstruction, cross-disciplinary, plant breeding, biobased economy

## Abstract

Despite significant progress toward the commercialization of biobased products, today’s biorefineries are far from achieving their intended goal of total biomass valorization and effective product diversification. The problem is conceptual. Modern biorefineries were built around well-optimized, cost-effective chemical synthesis routes, like those used in petroleum refineries for the synthesis of fuels, plastics, and solvents. However, these were designed for the conversion of fossil resources and are far from optimal for the processing of biomass, which has unique chemical characteristics. Accordingly, existing biomass commodities were never intended for modern biorefineries as they were bred to meet the needs of conventional agriculture. In this *perspective paper*, we propose a new path toward the design of efficient biorefineries, which capitalizes on a cross-disciplinary synergy between plant, physical, and catalysis science. In our view, the best opportunity to advance profitable and sustainable biorefineries requires the parallel development of novel feedstocks, conversion protocols and synthesis routes specifically tailored for total biomass valorization. Above all, we believe that plant biologists and process technologists can jointly explore the natural diversity of plants to synchronously develop both, biobased crops with designer chemistries and compatible conversion protocols that enable maximal biomass valorization with minimum input utilization. By building biorefineries from the bottom-up (i.e., starting with the crop), the envisioned partnership promises to develop cost-effective, biomass-dedicated routes which can be effectively scaled-up to deliver profitable and resource-use efficient biorefineries.

## Introduction

Climate change and the depletion of fossil reserves are decisive factors driving the development of a global biobased economy; that is, an economy that relies on renewable biomass for the concerted production of food, materials, chemicals, and energy. Prospectively, the replacement of fossil feedstocks with carbon-neutral alternatives can improve the energy, waste, and greenhouse gas balance of industrial production chains to address urgent societal needs (Ragauskas et al., 2006). A biobased economy therefore commands the wide-scale deployment of *integrated biorefineries* where biomass is entirely valorized to reduce waste and mitigate land use competition.

Agriculture needs to play a prominent role in the development of a sustainable biobased economy (Ragauskas et al., 2006; Perlack et al., 2011). For long, plants have been recognized as the *best chemists* on earth. They can capture sunlight and carbon dioxide to produce a plethora of molecules with unique and intricate functionalities; a great fraction of which cannot be replicated *via* industrial chemical synthesis. Thus, beyond food and feed, agriculture will continue to provide modern society with a wide array of industrial materials (*e.g.,* high-performance fibers), chemical commodities (*e.g.,* starch, rubber, oils), and specialty molecules (*e.g.,* taxol, opioids, artemisinin).

Remarkably, the vast potential of plant molecular diversity is yet to be harnessed in the context of a biobased economy. Until now, biorefineries have been conceptualized from the optic of chemists and are typically built around chemical synthesis routes which prioritize on the extraction and utilization of one specific biomass component (*e.g.,* cellulose from maize stems for the production of ethanol). This strategy comes with a major drawback. The highly heterogeneous and functionalized molecules of plants assemble hierarchically to form highly intertwined, recalcitrant structures (McCann and Carpita, 2015). Thus, biomass must undergo intensive physical and chemical disruption to liberate its constituent molecules; sometimes at the expense of the chemical integrity and quality of the final product, often at the expense of other molecules with great valorization potential (*e.g.,* lignin, hemicellulose, and protein residues from the production of ethanol from maize stems). It should thus become apparent that the broad spectrum of chemical functionalities available in agricultural and industrial crops remains vastly unexplored and underutilized in modern biorefineries.

So how can we get the most out of the molecular treasure trove available in the plant kingdom? In this *perspective paper*, we propose a new path toward the exploration and efficient valorization of plant molecular diversity in the context of a biobased economy. In our vision, plant scientists should immerse in the world of chemists and process technologists to gain new perspectives on the molecular and nanoscale forces that hold plants together, as well as to explore the natural diversity of plants in the search for novel chemistries and unimagined molecular structures. This cross-disciplinary synergy should lead to the rational utilization of plant molecular diversity in the creation of novel biobased crops with designer-chemistries that facilitate the separation and recovery of all valuable biomass components. Accordingly, the development of made-to-deconstruct feedstocks will lead way to compatible conversion protocols that enable maximal biomass valorization with minimum input utilization. Prospectively, the availability of dedicated biobased feedstocks and biomass-specific conversion routes will help us reimagine modern biorefineries in favor of more profitable and resource-use efficient models.

## Biomass Deconstruction: The Real Hurdle Toward Total-Use of Biomass Biorefineries

Biomass-process engineers are well aware that to maximize the sustainability and cost efficiency of biorefineries, they must separate all components out of biomass in their highest possible value (i.e., by preserving wherein possible their original chemical structure and functionality) using the lowest possible amount of chemical and energetic inputs (i.e., leading to inefficient exergy loss). Theoretically, this strategy would enable them access to the full spectrum of chemical functionalities naturally present in plants and open new opportunities for biobased product innovation and diversification (Gallezot, 2012; Dusselier et al., 2014).

In this regard, the best opportunity for the industry to achieve its sustainability goals lies on designing effective biomass deconstruction technologies (**BDTs**), which can efficiently disentangle and liberate biomass polymers and active compounds with lowest loss of resources. Today, **BDTs** are considered the most important cost deterrents in the industry (Wyman, 2007; McCann and Carpita, 2015), and life-cycle assessments repeatedly demonstrate that **BDTs** contribute significantly to the unsustainable consumption of water, energy, and chemicals to advanced biorefineries (Tao et al., 2013). Therefore, in the last decade, significant efforts have been devoted to the development of scalable, cost-competitive, and sustainable biomass deconstruction technologies. Irrespective of biorefinery application (i.e., food, feed, fuels, and chemicals) or approach (i.e., physical, chemical, and biological), these efforts have focused on reducing the material and energy requirements needed to “crack” biomass into simpler molecular species. Notable advances include the development of dry fractionation (Barakat et al., 2014; Van Der Goot et al., 2016), mild-solvent fractionation (Konda et al., 2014; Luterbacher et al., 2014; Socha et al., 2014), bio-catalysis (Linger et al., 2014), and consolidated bioprocessing technologies (Xu et al., 2016). More disruptive technologies are also beginning to gain ground; recent achievements include, for instance, the employment of microwaves (Liu et al., 2018) and ultra-sound-assisted fractionation to separate cellulose and hemicellulose form lignocellulosic biomass. Despite fundamental breakthroughs, the economic and environmental burden of disassembling recalcitrant biomass still remains the main challenge in the industry, but we believe that with a change in paradigm, this problem opens unwarranted opportunities.

## Redesigning Biorefineries from the Bottom-Up, Starting with the Feedstock

From the optic of chemists and process engineers, the contribution of plant scientists to the development of sustainable biorefineries is primarily left to the identification of plant materials that can be produced inexpensively and in abundant quantities. This vision derives from techno-economic assessments, which indicate that the final cost of biobased products is sensitive to fluctuations in the price and local availability of agricultural feedstocks (Wright and Brown, 2007; Kim and Dale, 2015). It also follows affirmations that parameters relevant to the sustainable production of biomass exert a critical impact on the environmental performance of biomass conversion technologies (Brehmer et al., 2009; Azapagic, 2014) and biorefineries (Garofalo et al., 2018).

Notwithstanding, plant scientists can also lead the way forward in addressing the industry’s greatest obstacle: the recalcitrance of biomass to efficient deconstruction. Today, plant breeders and molecular biologists are developing strategies to enable the modification *in planta* of the composition, structure, and bioavailability of valuable plant components (i.e., polymers, metabolites, etc.) so that these require lower energetic and chemical inputs for effective extraction and/or chemical conversion (Box 1). A flagship example comes from cellulosic fuel refineries, where lignin constitutes a technical barrier to the effective depolymerization of the structural carbohydrates (i.e., cellulose and hemicellulose) of lignocellulose (Torres et al., 2015, 2016). Extensive genetic surveys have demonstrated that biomass lignification is a highly heritable trait which can be selected against without incurring a significant penalty on plant yield (McCann and Carpita, 2015; Torres et al., 2015). This has enabled the development of highly productive, low-lignin biomass cultivars, which can be deconstructed using low-severity thermochemical and enzymatic processing to release competitive fermentable sugar yields (McCann and Carpita, 2015; Torres et al., 2016). In fact, quantitative analyses show that bioprocessing of these feedstocks can significantly improve the economic performance and environmental footprint of lignocellulose-to-ethanol conversion pathways (Torres et al., 2016).

BOX 1 **The untapped potential of plant species**

Crops grown today are far from realizing the technical demands of modern biorefineries, as they have been purposely bred to accommodate traditional agricultural and industrial models. Notwithstanding, as of today, the vast genetic diversity present in agricultural crops remains largely unexplored. Within the same species, this diversity can extend from macroscopic characters (e.g., plant anatomical architecture) to molecular-level differences in the composition, structure, and localization of biopolymers (McCann and Carpita, 2015; Simmons et al., 2016). By mining this natural genetic diversity, ample opportunities exist to construct new crops, with specific chemical structures, which complement the mode of action of biomass deconstruction and depolymerization processes. Accordingly, the latest developments in genetic engineering technologies and synthetic biology are enabling us to tailor the chemical, physical, and rheological properties of plant materials beyond their natural boundaries. Fundamental breakthroughs in this direction include the incorporation of novel chemistries in biomass to facilitate its disassembly (Zhang et al., 2012; Wilkerson et al., 2014), the integration of catalysts which guide the self-deconstruction of biomass feedstocks (Shen et al., 2012; Giessen and Silver, 2016; Donohoe et al., 2017), and metabolic pathway compartmentalization for increased production of valuable metabolites and ease of separation. To illustrate, Lin et al. (2016) recently developed an elegant transgenic strategy to express the iron-storage protein, ferritin, in the model plant species, Arabidopsis thaliana. Transformed plants presented improved enzymatic digestibility of lignocellulose following mild dilute-acid pretreatment. This improvement was ascribed to the action of accumulated iron ions acting as accessory co-catalysts in the degradation of cell wall chemical bonds during pretreatment. Understandably, while this and other studies open new routes for the incorporation of deconstruction catalysts and labile bonds in plant structures, further developments in the creation of “labile” feedstocks (whether through classical breeding or genetic engineering) will be confined by detrimental effects on plant health and productivity that can derive from these interventions.

**Figure 1:**
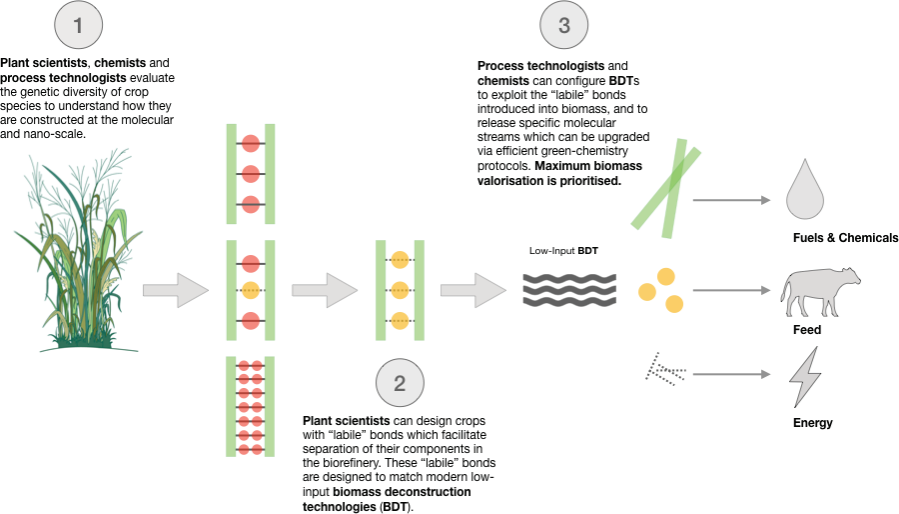
Conceptual workflow toward the design of modern biorefineries from the bottom-up, starting from the feedstock.

The development of feedstocks with reduced recalcitrance can facilitate the advance of selective and less intensive process configurations. But to effectively maximize resource-use efficiency in the biorefinery, plant scientists must overcome two interrelated challenges. On one side, future biobased crops need to be designed as specialized *biofactories*, which can simultaneously generate a wide range of usable products (i.e., carbohydrates, proteins, lipids, and secondary metabolites) with desired properties and functionalities. On the other side, these feedstocks must exhibit ease of dissociation under low-impact bioprocessing to guarantee the effective extraction of all available biomass components in their highest value. Plant scientists therefore have the herculean task of redesigning crops beyond “single-product” commodity systems (Box 1) to deliver a new generation of agricultural feedstocks specifically designed to facilitate total biomass valorization. This task demands a fundamental departure from conventional plant breeding, but it opens an unprecedented opportunity for the rational design of modern biorefineries from the bottom-up, starting from the feedstock (Figure 1).

## Creating Novel Biorefinery Concepts Through a Cross-Disciplinary Synergies

Chemists and process technologists are currently focused on gaining a molecular-level understanding of the physical-chemical interactions and forces that bind biomolecules and prohibit biomass disassembly and depolymerization processes. Aided by the latest advances in computational modeling (Silveira et al., 2014; Sun et al., 2014; Pereira et al., 2017), high-resolution imaging (Selig et al., 2007; Cheng et al., 2015; Donaldson and Vaidya, 2017; Haarmeyer et al., 2017), nano-mechanic analysis (Farahi et al., 2017), and chemo-analytical instrumentation (Simmons et al., 2016; Kang et al., 2019), they have demonstrated that biological polymers conform supramolecular structures whose “lability” is governed by physicochemical phenomena. During conversion, biological structures can be dissociated and transformed into a wide array of simple and aggregated molecular species; many of which exhibit unsuspected chemistries and topologies that adversely affect the efficiency of biomass disassembly and upstream conversion protocols (Selig et al., 2007; Cheng et al., 2015; Haarmeyer et al., 2017).

By jointly exploring the genetic diversity of crop species at the molecular- and nano-scale, plant scientists, chemists, and process engineers can gain radically novel insights into the extensive constellation of supramolecular structures available in the plant kingdom, as well as information on how these structures impact the selectivity and yield of biomass conversion processes. This inventoried knowledge can be used to rationally design and specify the molecular species and interactions within biomass that yield desired chemical products under specific conversion strategies (Box 2; Nordwald et al., 2014; McCann and Carpita, 2015; Whitehead et al., 2017). Essentially, plant breeders will obtain design guidelines to fine-tune the content and chemistry of biological polymers to create molecular complexes which are sufficiently robust to sustain plant life, but amenable to deconstruction under low-impact bioprocessing. Accordingly, an understanding of the physicochemical forces that control the association of these complexes will enable deconstruction scientists to focus on the directed design of separation configurations that match new biomolecule interactions. Novel biomass deconstruction technologies, will also need to be intelligently designed and aligned with the ideas of catalysis experts (Bussemaker and Zhang, 2013), so that their outputs derive into chemical streams which can be effectively upgraded *via* efficient green-chemistry protocols. In the long run, the envisioned collaboration between plant, chemistry, and process experts enables the development of synergistic innovations (from seed to end-product) which collectively conform resource-use efficient biomass conversion routes that can be scaled up to deliver profitable and sustainable biorefineries (Figure 1).

BOX 2 **A conceptual synergy in the starch biorefinery**

Existing starch biorefineries can be regarded as a prototype for our synergistic biorefinery concept. In the past decades, the development of viable and sustainable starch biorefineries has been propelled by the establishment and development of joint efforts between plant scientists and process engineers. This unique synergy has enabled the systematic design of technological processes adapted to novel starches. In this integrated framework, plant scientists have made considerable efforts to modify starches with enhanced characteristics in planta aiming for efficient and effective processes (Xu et al., 2014), and process technologists have simultaneously optimized process systems that generate novel starches.

One of the successful examples comes from producing mono- or disaccharide sugars from starch in the industry (Hebelstrup et al., 2015). Instead of enzymatic starch hydrolysis in the processing tank, the hydrolytic processes are proceeded in the plants by expression of hydrolytic enzymes in crop organs (Santa-Maria et al., 2011). These “self-processing” plants accumulate hydrolytic enzymes during plant development without detriment to plant viability. Starch hydrolysis is activated during industrial processing by turning on the “control switch,” such as heat treatment, that was embedded into the starch-storing organs using biotechnology. Such designs have been achieved in many starch crops (Beaujean et al., 2000; Xu et al., 2008; Santa-Maria et al., 2011) and integrated smoothly into the existing infrastructure (Urbanchuk et al., 2009). It was reported that self-processing corn can largely reduce the production costs and energy and water usage of processing (Urbanchuk et al., 2009). Another remarkable invention is starch-based biodegradable bioplastic Biolice^®^,^1^ which embodies an integrative product of plant breeding and processing technology innovation. In this novel process, granules are directly extruded from corn flour rather than going through an energy-consuming and costly starch extraction step. Clearly, innovations towards a sustainable starch biorefinery demand concerted and sustained efforts from both plant scientists and biorefinery scientists.

**Figure 2:**
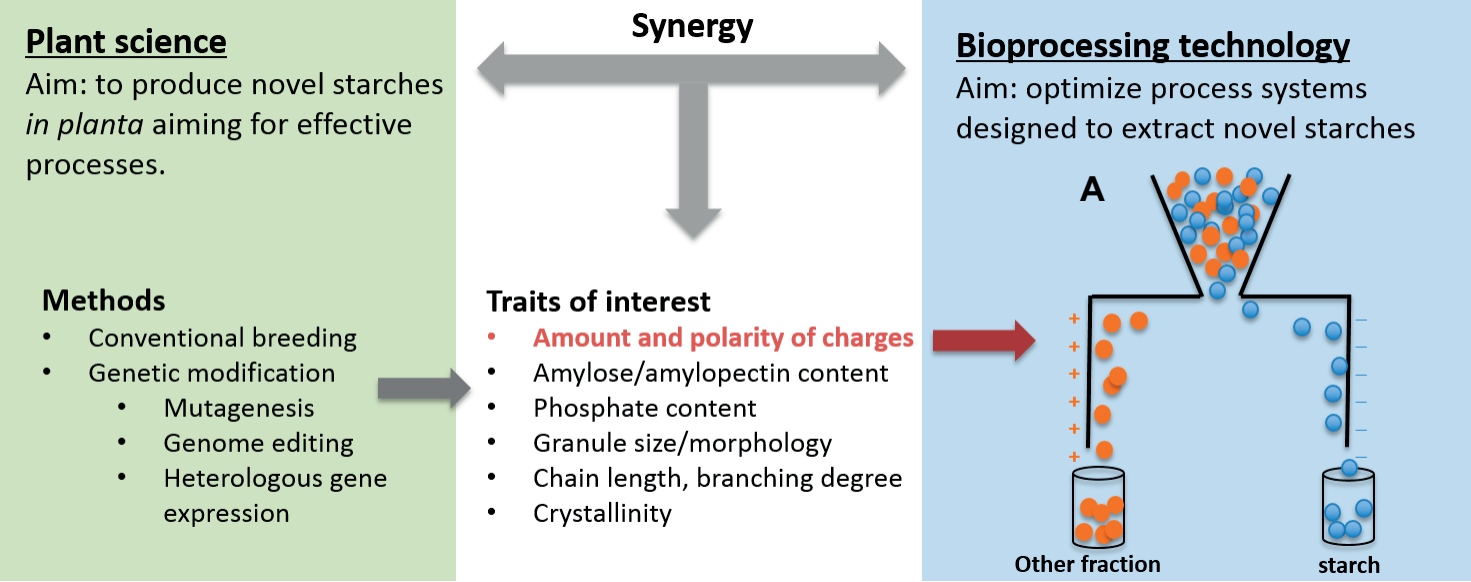
Synergy between plant sciences and bioprocessing technologies in starch biorefinery, with focus on (A) the design of electrostatic separation process.

Tremendous opportunities for innovation are ahead in the example of starch biorefinery if we further enhance this synergy to create systematic designs (Figure 2). In fact, with the increasing availability of plant breeding and biotechnological tools, plant scientists are gaining greater capabilities to design fit-for-process starches, thus facilitating innovative processes (Xu et al., 2014). Currently, processing scientists are developing an electrostatic separation process for dry separation of food ingredients (e.g., starch, protein, fiber etc.) (Wang et al., 2014, 2015, 2016). This sustainable process separates different ingredients/particles in an external electric field by initially charging the powder particles. Separation efficiency depends highly on the polarity of the particles and the sufficiency of charges (Wang et al., 2014, 2015). To this regard, we envisage in planta synthesizing novel starches that carry a defined amount and/or polarity of charges by introducing/removing different charged groups, thus enhancing the dispersibility of the mixtures. This could be achieved through plant breeding program and/or biotechnological approach. For instance, in order to alter the amount of negative charge of starch granules, plant scientists can add or remove starch-attached phosphate groups (charged negatively) by manipulating the expression of key genes involved in starch biosynthesis (Lanahan and Basu, 2006; Schewe et al., 2007; Carciofi et al., 2011; Frohberg, 2012; Xu et al., 2017a,b, 2018). We firmly believe that such design can be realized through joint efforts from plant scientists and biorefinery scientist and will play an important role in elevating the starch biorefinery to new heights.

## Concluding Remarks: The Path Forward

Without doubt, the challenge of developing profitable and sustainable biorefineries is a complex problem that requires a multifocal solution. This challenge is being currently addressed from different scientific fronts but must be vehement on the fact that a knowledge barrier exists between the participating disciplines. Thus far, while process scientists have been exploring new methods to disassemble unyielding plant materials, plant scientists have been designing labile crops for conventional biorefinery concepts which do not fulfill the sustainability goals envisioned for the industry. In essence, the most promising and ground-breaking innovations from both worlds are currently redundant or misguided.

The current state of affairs highlights the critical need for the establishment of “true” integrative workflows between plant scientists, chemists, and process experts. The proposed synergy would need to extend beyond unidirectional collaborations, where the dogmatic views of one discipline prevail over others, to support the creation of multidisciplinary teams which jointly conceptualize, design, and validate biorefinery technologies. These collaborative networks should enable a continuous flow of concepts, methodologies and resources across disciplines to guide non-redundant technological improvements aligned with the real needs of the industry.

In the long run, the synergistic design of biorefineries will lead to the production of integrated solution packages (i.e., novel feedstocks and processes that match each other). The sum of these complimentary scientific interventions is likely to yield greater benefits than isolated and unaligned solutions from both scientific fronts. This concept, of course, is not completely new; the marriage between plant breeding and biorefinery science is the basis for the commercial success of a few biobased technologies (Box 2). It is also driving current efforts toward the design of effective biorefineries for the production of cellulosic fuels and platform chemicals. In the backdrop of these developments, our proposition is that these multifocal efforts become a ubiquitous aspect of academic and industrial efforts working on the design, implementation and commercialization of biorefinery technologies.

## Author Contributions

AT, LT, CN and JB conceptualized the main idea of this manuscript. AT and XX wrote the manuscript. All authors reviewed and approved the manuscript.

### Conflict of Interest Statement

The authors declare that the research was conducted in the absence of any commercial or financial relationships that could be construed as a potential conflict of interest.

## Footnotes

^1^www.lci.limagrain.com

## References

[ref1] Azapagic A. (2014). Sustainability considerations for integrated biorefineries. Trends Biotechnol. 32, 1–4. 10.1016/j.tibtech.2013.10.009, PMID: 24364880

[ref2] Barakat A. Chuetor S. Monlau F. Solhy A. Rouau X. (2014). Eco-friendly dry chemo-mechanical pretreatments of lignocellulosic biomass: impact on energy and yield of the enzymatic hydrolysis. Appl. Energy 113, 97–105. 10.1016/j.apenergy.2013.07.015

[ref3] Beaujean A. Ducrocq-Assaf C. Sangwan R. Lilius G. Bülow L. Sangwan-Norreel B. (2000). Engineering direct fructose production in processed potato tubers by expressing a bifunctional alpha-amylase/glucose isomerase gene complex. Biotechnol. Bioeng. 70, 9–16. 10.1002/1097-0290(20001005)70:1<9::AID-BIT2>3.0.CO;2-7, PMID: 10940858

[ref4] Brehmer B. Boom R. M. Sanders J. (2009). Maximum fossil fuel feedstock replacement potential of petrochemicals via biorefineries. Chem. Eng. Res. Des. 87, 1103–1119. 10.1016/j.cherd.2009.07.010

[ref5] Bussemaker M. J. Zhang D. (2013). Effect of ultrasound on lignocellulosic biomass as a pretreatment for biorefinery and biofuel applications. Ind. Eng. Chem. Res. 52, 3563–3580. 10.1021/ie3022785

[ref6] Carciofi M. Shaik S. S. Jensen S. L. Blennow A. Svensson J. T. Vincze É. (2011). Hyperphosphorylation of cereal starch. J. Cereal Sci. 54, 339–346. 10.1016/j.jcs.2011.06.013

[ref7] Cheng G. Zhang X. Simmons B. Singh S. (2015). Theory, practice and prospects of X-ray and neutron scattering for lignocellulosic biomass characterization: towards understanding biomass pretreatment. Energy Environ. Sci. 8, 436–455. 10.1039/C4EE03147D

[ref8] Donaldson L. Vaidya A. (2017). Visualising recalcitrance by colocalisation of cellulase, lignin and cellulose in pretreated pine biomass using fluorescence microscopy. Sci. Rep. 7:44386. 10.1038/srep44386, PMID: 28281670 PMC5345003

[ref9] Donohoe B. S. Wei H. Mittal A. Shollenberger T. Lunin V. V. Himmel M. E. (2017). Towards an understanding of enhanced biomass digestibility by in planta expression of a family 5 glycoside hydrolase. Sci. Rep. 7:4389. 10.1038/s41598-017-04502-1 28663545 PMC5491509

[ref10] Dusselier M. Mascal M. Sels B. F. (2014). “Top chemical opportunities from carbohydrate biomass: a chemist’s view of the biorefinery” in Selective Catalysis for Renewable Feedstocks and Chemicals. eds. Nicholas K. (Cham: Springer), 1–40. 10.1007/128_2014_544 24842622

[ref11] Farahi R. Charrier A. M. Tolbert A. Lereu A. L. Ragauskas A. Davison B. H. (2017). Plasticity, elasticity, and adhesion energy of plant cell walls: nanometrology of lignin loss using atomic force microscopy. Sci. Rep. 7:152. 10.1038/s41598-017-00234-4 28273953 PMC5428038

[ref12] Frohberg C. (2012). *Genetically modified plants which synthesize a starch having increased swelling power*. Google Patents.

[ref13] Gallezot P. (2012). Conversion of biomass to selected chemical products. Chem. Soc. Rev. 41, 1538–1558. 10.1039/C1CS15147A, PMID: 21909591

[ref14] Garofalo P. Campi P. Vonella A. V. Mastrorilli M. (2018). Application of multi-metric analysis for the evaluation of energy performance and energy use efficiency of sweet sorghum in the bioethanol supply-chain: a fuzzy-based expert system approach. Appl. Energy 220, 313–324. 10.1016/j.apenergy.2018.03.065

[ref15] Giessen T. W. Silver P. A. (2016). Encapsulation as a strategy for the design of biological compartmentalization. J. Mol. Biol. 428, 916–927. 10.1016/j.jmb.2015.09.009, PMID: 26403362

[ref16] Haarmeyer C. N. Smith M. D. Chundawat S. P. S. Sammond D. Whitehead T. A. (2017). Insights into cellulase-lignin non-specific binding revealed by computational redesign of the surface of green fluorescent protein. Biotechnol. Bioeng. 114, 740–750. 10.1002/bit.26201, PMID: 27748522

[ref17] Hebelstrup K. H. Sagnelli D. Blennow A. (2015). The future of starch bioengineering: GM microorganisms or GM plants? Front. Plant Sci. 6:247. 10.3389/fpls.2015.00247, PMID: 25954284 PMC4407504

[ref18] Kang X. Kirui A. Widanage M. C. D. Mentink-Vigier F. Cosgrove D. J. Wang T. (2019). Lignin-polysaccharide interactions in plant secondary cell walls revealed by solid-state NMR. Nat. Commun. 10:347. 10.1038/s41467-018-08252-0 30664653 PMC6341099

[ref19] Kim S. Dale B. E. (2015). All biomass is local: the cost, volume produced, and global warming impact of cellulosic biofuels depend strongly on logistics and local conditions. Biofuels Bioprod. Biorefin. 9, 422–434. 10.1002/bbb.1554

[ref20] Konda N. M. Shi J. Singh S. Blanch H. W. Simmons B. A. Klein-Marcuschamer D. (2014). Understanding cost drivers and economic potential of two variants of ionic liquid pretreatment for cellulosic biofuel production. Biotechnol. Biofuels 7:86. 10.1186/1754-6834-7-86, PMID: 24932217 PMC4055852

[ref21] Lanahan M. Basu S. (2006). *Modified starch, uses, methods for production thereof*. Google Patents.

[ref22] Lin C.-Y. Jakes J. E. Donohoe B. S. Ciesielski P. N. Yang H. Gleber S.-C. (2016). Directed plant cell-wall accumulation of iron: embedding co-catalyst for efficient biomass conversion. Biotechnol. Biofuels 9:225. 10.1186/s13068-016-0639-2 27777626 PMC5073452

[ref23] Linger J. G. Vardon D. R. Guarnieri M. T. Karp E. M. Hunsinger G. B. Franden M. A. (2014). Lignin valorization through integrated biological funneling and chemical catalysis. Proc. Natl. Acad. Sci. USA 111, 12013–12018. 10.1073/pnas.1410657111 25092344 PMC4143016

[ref24] Liu Y. Sun B. Zheng X. Yu L. Li J. (2018). Integrated microwave and alkaline treatment for the separation between hemicelluloses and cellulose from cellulosic fibers. Bioresour. Technol. 247, 859–863. 10.1016/j.biortech.2017.08.059, PMID: 30060423

[ref25] Luterbacher J. S. Rand J. M. Alonso D. M. Han J. Youngquist J. T. Maravelias C. T.. (2014). Nonenzymatic sugar production from biomass using biomass-derived γ-valerolactone. Science 343, 277–280. 10.1126/science.1246748, PMID: 24436415

[ref26] McCann M. C. Carpita N. C. (2015). Biomass recalcitrance: a multi-scale, multi-factor, and conversion-specific property. J. Exp. Bot. 66, 4109–4118. 10.1093/jxb/erv267, PMID: 26060266

[ref27] Nordwald E. M. Brunecky R. Himmel M. E. Beckham G. T. Kaar J. L. (2014). Charge engineering of cellulases improves ionic liquid tolerance and reduces lignin inhibition. Biotechnol. Bioeng. 111, 1541–1549. 10.1002/bit.25216, PMID: 24522957

[ref28] Pereira C. S. Silveira R. L. Dupree P. Skaf M. S. (2017). Effects of xylan side-chain substitutions on xylan–cellulose interactions and implications for thermal pretreatment of cellulosic biomass. Biomacromolecules 18, 1311–1321. 10.1021/acs.biomac.7b00067, PMID: 28252951

[ref29] Perlack R. D. Eaton L. M. Turhollow A. F. Jr. Langholtz M. H. Brandt C. C. Downing M. E. (2011). US billion-ton update: biomass supply for a bioenergy and bioproducts industry. Oak Ridge, TN: Oak Ridge National Laboratory, p 227.

[ref30] Ragauskas A. J. Williams C. K. Davison B. H. Britovsek G. Cairney J. Eckert C. A.. (2006). The path forward for biofuels and biomaterials. Science 311, 484–489. 10.1126/science.1114736, PMID: 16439654

[ref31] Santa-Maria M. C. Yencho C. G. Haigler C. H. Thompson W. F. Kelly R. M. Sosinski B. (2011). Starch self-processing in transgenic sweet potato roots expressing a hyperthermophilic α-amylase. Biotechnol. Prog. 27, 351–359. 10.1002/btpr.573, PMID: 21365786

[ref32] Schewe G. Knies P. Amati S. F. Lörz H. Becker D. Landschütze V. (2007). *Monocotyledon plant cells and plants which synthesise modified starch*. Google Patents.

[ref33] Selig M. J. Viamajala S. Decker S. R. Tucker M. P. Himmel M. E. Vinzant T. B. (2007). Deposition of lignin droplets produced during dilute acid pretreatment of maize stems retards enzymatic hydrolysis of cellulose. Biotechnol. Prog. 23, 1333–1339. 10.1021/bp0702018, PMID: 17973399

[ref34] Shen B. Sun X. Zuo X. Shilling T. Apgar J. Ross M.. (2012). Engineering a thermoregulated intein-modified xylanase into maize for consolidated lignocellulosic biomass processing. Nat. Biotechnol. 30:1131. 10.1038/nbt.2402, PMID: 23086202

[ref35] Silveira R. L. Stoyanov S. R. Gusarov S. Skaf M. S. Kovalenko A. (2014). Supramolecular interactions in secondary plant cell walls: effect of lignin chemical composition revealed with the molecular theory of solvation. J. Phys. Chem. Lett. 6, 206–211. 10.1021/jz502298q 26263115

[ref36] Simmons T. J. Mortimer J. C. Bernardinelli O. D. Pöppler A.-C. Brown S. P. Dupree R.. (2016). Folding of xylan onto cellulose fibrils in plant cell walls revealed by solid-state NMR. Nat. Commun. 7:13902. 10.1038/ncomms13902, PMID: 28000667 PMC5187587

[ref37] Socha A. M. Parthasarathi R. Shi J. Pattathil S. Whyte D. Bergeron M.. (2014). Efficient biomass pretreatment using ionic liquids derived from lignin and hemicellulose. Proc. Natl. Acad. Sci. USA 111, E3587–E3595. 10.1073/pnas.1405685111, PMID: 25136131 PMC4156760

[ref38] Sun N. Parthasarathi R. Socha A. M. Shi J. Zhang S. Stavila V. (2014). Understanding pretreatment efficacy of four cholinium and imidazolium ionic liquids by chemistry and computation. Green Chem. 16, 2546–2557. 10.1039/C3GC42401D

[ref39] Tao L. Tan E. C. Aden A. Elander R. T. (2013). “Techno-economic analysis and life-cycle assessment of lignocellulosic biomass to sugars using various pretreatment technologies” in Biological Conversion of Biomass for Fuels and Chemicals. eds. Sun J. Ding S.-Y. Peterson J. D. (Oxfordshire: The Royal Society of Chemistry), 358–380.

[ref40] Torres A. F. Slegers P. M. Noordam-Boot C. M. Dolstra O. Vlaswinkel L. Boxtel A. J. (2016). Maize feedstocks with improved digestibility reduce the costs and environmental impacts of biomass pretreatment and saccharification. Biotechnol. Biofuels 9:63. 10.1186/s13068-016-0479-0 26981155 PMC4791978

[ref41] Torres A. F. Visser R. G. Trindade L. M. (2015). Bioethanol from maize cell walls: genes, molecular tools, and breeding prospects. GCB Bioenergy 7, 591–607. 10.1111/gcbb.12164

[ref42] Urbanchuk J. M. Kowalski D. J. Dale B. Kim S. (2009). Corn amylase: improving the efficiency and environmental footprint of corn to ethanol through plant biotechnology. AgBioForum 12, 149–154.

[ref43] Van Der Goot A. J. Pelgrom P. J. Berghout J. A. Geerts M. E. Jankowiak L. Hardt N. A. (2016). Concepts for further sustainable production of foods. J. Food Eng. 168, 42–51. 10.1016/j.jfoodeng.2015.07.010

[ref44] Wang J. De Wit M. Boom R. M. Schutyser M. A. (2015). Charging and separation behavior of gluten–starch mixtures assessed with a custom-built electrostatic separator. Sep. Purif. Technol. 152, 164–171. 10.1016/j.seppur.2015.08.025

[ref45] Wang J. De Wit M. Schutyser M. A. Boom R. M. (2014). Analysis of electrostatic powder charging for fractionation of foods. Innovative Food Sci. Emerg. Technol. 26, 360–365. 10.1016/j.ifset.2014.06.011

[ref46] Wang J. Zhao J. De Wit M. Boom R. M. Schutyser M. A. (2016). Lupine protein enrichment by milling and electrostatic separation. Innovative Food Sci. Emerg. Technol. 33, 596–602. 10.1016/j.ifset.2015.12.020

[ref47] Whitehead T. A. Bandi C. K. Berger M. Park J. Chundawat S. P. (2017). Negatively supercharging cellulases render them lignin-resistant. ACS Sustain. Chem. Eng. 5, 6247–6252. 10.1021/acssuschemeng.7b01202

[ref48] Wilkerson C. Mansfield S. Lu F. Withers S. Park J.-Y. Karlen S.. (2014). Monolignol ferulate transferase introduces chemically labile linkages into the lignin backbone. Science 344, 90–93. 10.1126/science.1250161, PMID: 24700858

[ref49] Wright M. Brown R. C. (2007). Establishing the optimal sizes of different kinds of biorefineries. Biofuels, Bioprod. Biorefin. 1, 191–200. 10.1002/bbb.25

[ref50] Wyman C. E. (2007). What is (and is not) vital to advancing cellulosic ethanol. Trends Biotechnol. 25, 153–157. 10.1016/j.tibtech.2007.02.009, PMID: 17320227

[ref51] Xu X. Dees D. Dechesne A. Huang X.-F. Visser R. G. Trindade L. M. (2017a). Starch phosphorylation plays an important role in starch biosynthesis. Carbohydr. Polym. 157, 1628–1637. 10.1016/j.carbpol.2016.11.043 27987877

[ref52] Xu X. Dees D. Huang X. F. Visser R. G. Trindade L. M. (2018). Heterologous expression of two Arabidopsis starch dikinases in potato. Starch-Stärke 70:1600324. 10.1002/star.201600324

[ref53] Xu X. Fang J. Wang W. Guo J. Chen P. Cheng J.. (2008). Expression of a bacterial α-amylase gene in transgenic rice seeds. Transgenic Res. 17, 645–650. 10.1007/s11248-007-9144-5, PMID: 17926139

[ref54] Xu X. Huang X.-F. Visser R. G. Trindade L. M. (2017b). Engineering potato starch with a higher phosphate content. PLoS One 12:e0169610. 10.1371/journal.pone.0169610 28056069 PMC5215930

[ref55] Xu F. Sun J. Konda N. M. Shi J. Dutta T. Scown C. D. (2016). Transforming biomass conversion with ionic liquids: process intensification and the development of a high-gravity, one-pot process for the production of cellulosic ethanol. Energy Environ. Sci. 9, 1042–1049. 10.1039/C5EE02940F

[ref56] Xu X. Visser R. G. Trindade L. M. (2014). “Starch modification by biotechnology: state of art and perspectives” in Starch Polymers: From Genetic Engineering to Green Applications. eds. Halley P. Avérous L. (Oxford: Elsevier), 79–102.

[ref57] Zhang K. Bhuiya M.-W. Pazo J. R. Miao Y. Kim H. Ralph J. (2012). An engineered monolignol 4-O-methyltransferase depresses lignin biosynthesis and confers novel metabolic capability in Arabidopsis. Plant Cell. 24, 3135–3152. 10.1105/tpc.112.101287 22851762 PMC3426137

